# A Rare Symptomatic Case of Congenital Origin of Right Coronary Artery From Left Coronary Sinus

**DOI:** 10.7759/cureus.25358

**Published:** 2022-05-26

**Authors:** Abu Baker Khan, Fatima Iqbal, Maryam Gul, Saad Ahmad, Mateen Ahmad

**Affiliations:** 1 Surgery, District Headquarter Hospital, Dera Ismail Khan, PAK; 2 Medicine, Type D Hospital Sub Division Darazinda, Dera Ismail Khan, PAK; 3 Internal Medicine, Taj Medical Center, Nowshera, PAK; 4 Surgery, Khyber Teaching Hospital, Peshawar, PAK

**Keywords:** surgical revascularization, angiography, myocardial infarction, coronary artery anomaly, congenital heart disease

## Abstract

An anomalous origin of the right coronary artery from the left coronary sinus is a rare congenital disorder, characterized by an asymptomatic presentation and an increased risk of myocardial infarction, arrhythmias, and sudden cardiac death. This disorder with an inter arterial course of the right coronary artery is subject to mechanical compression leading to various symptoms. Only a handful of studies are published related to the atypical origin of coronary arteries. Therefore, we present a case of a hospitalized adult diagnosed with an atypical origin of the right coronary artery from the left coronary sinus. A 51-year-old female presented with mid-sternal heaviness, pressure, and burning sensation, not accompanied by sweating, dizziness, or light-headedness. Biochemical studies revealed an elevated troponin 1 level of 0.12 ng/mL. A coronary arteriogram showed proximal stenosis of the right coronary artery. CT cardiac angiography revealed a large right coronary artery arising from the left cusp anterior to the left main coronary artery. The patient was treated with surgical revascularization therapy.

## Introduction

An atypical origin of the right coronary artery from the left coronary sinus is a rare congenital anomaly accounting for 0.1% of coronary angiography patients [1]. After originating from the left coronary cusp, the right coronary artery most often follows an inter-arterial course between the aorta and the pulmonary artery to its usual destination. Due to this aberrant course, it is subjective to mechanical compression between the great vessels and can present with a wide range of symptoms [2]. Although the literature shows that most of these patients are clinically asymptomatic, patients can present with angina, shortness of breath, and syncope. Myocardial infarction, arrhythmias, and sudden cardiac death in the absence of atherosclerotic heart disease are major risk factors [3]. The management strategies vary from case to case depending upon the subtypes of anomalies; however, recent surgical treatment is gaining the spotlight for managing patients at high risk of sudden cardiac death [4].

## Case presentation

On September 2, 2021, a 51-year-old white female was hospitalized in the emergency department. She presented with the third episode of mid-sternal chest heaviness and pressure, which radiate to her left arm. She also complained of heaviness in her left arm and a burning sensation in the mid-retrosternal region, not accompanied by sweating, shortness of breath, dizziness, or light-headedness. The history of lower chest obstruction and difficulty swallowing was positive for two weeks. She considered that the symptoms were the result of gastroesophageal reflux. According to the patient, one night, she did not have relief throughout the night, followed by a similar episode last night when she was on a nitroglycerine infusion. The patient used to smoke one pack of cigarettes daily for the past 20 years. She had a positive history of arterial hypertension and antihypertensive medications. She had a significant family history of coronary heart disease with her father undergoing coronary artery bypass grafting. One of her brothers is hypertensive with a high cholesterol level.

Her vitals on initial presentation were as follows - blood pressure (BP): 135/85, pulse rate: 92 beats per minute, respiratory rate: 17 breaths per minute, and pulse oximetry: 97%. Physical examination was unremarkable. The electrocardiogram showed normal sinus rhythm with no ST-segment and T-wave abnormalities. Laboratory investigations revealed an elevated troponin 1 level (0.12 ng/mL), accounting for non-Q wave myocardial infarction.

**Figure 1:**
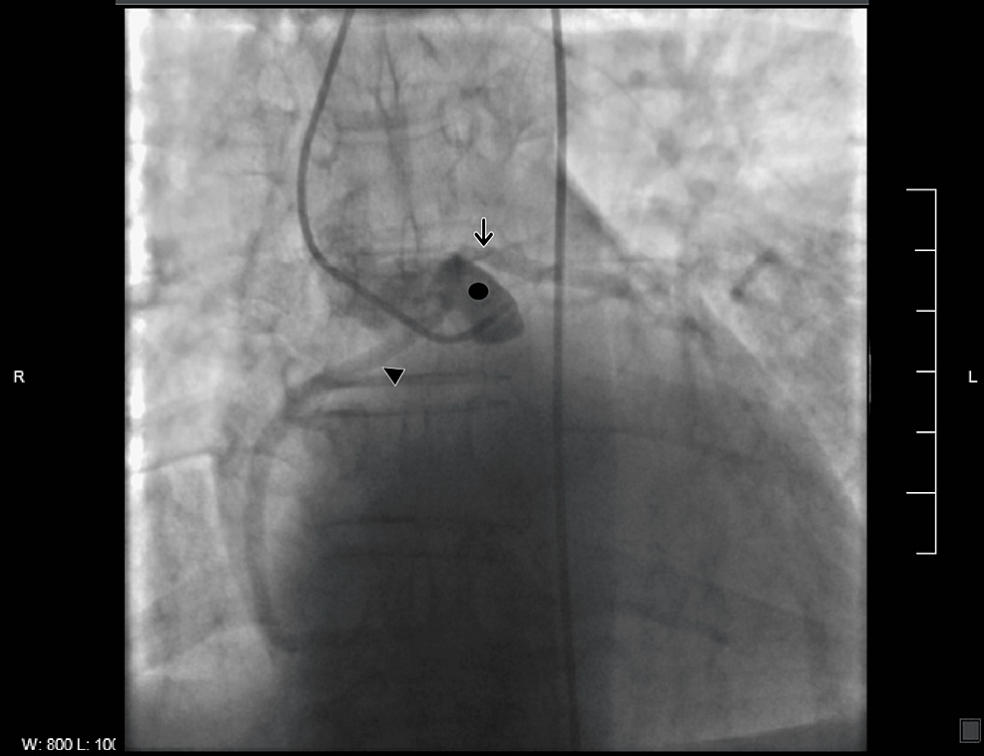
Coronary angiography showing origin of both right coronary artery (arrowhead 🢐) and left coronary artery (arrow 🠗) from left coronary sinus (solid circle ●).

Due to a constellation of clinical symptoms, laboratory findings, and a strong family history of coronary heart disease, the patient was taken to a catheterization laboratory and a coronary arteriogram was performed. On coronary angiogram, the left main coronary artery was moderate to large with minimal ostial tapering and no significant obstruction thereafter. The right coronary artery was difficult to cannulate with multiple catheters, which eventually demonstrated the right coronary artery originating from the left coronary cusp adjacent to the left main coronary artery (Figure 1).

**Figure 2:**
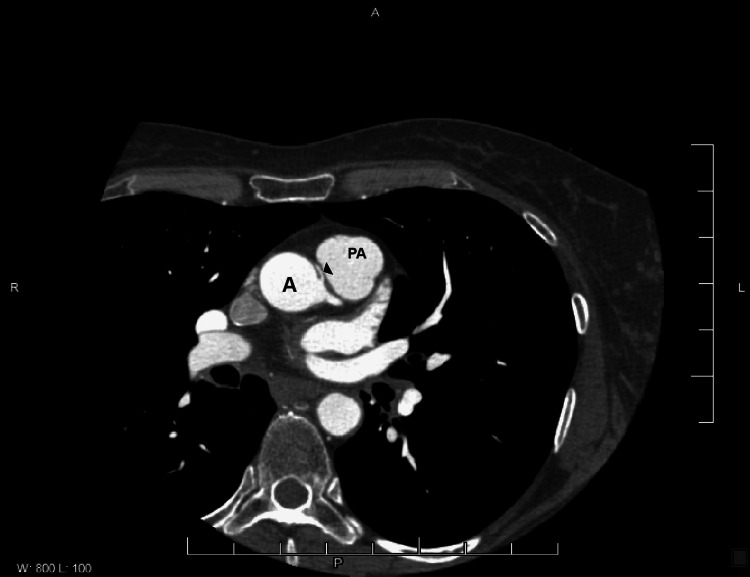
CT cardiac angiography showing anomalous origin of right coronary artery from left cusp (arrowhead 🢐). The RCA then exhibits an inter-arterial course between the aorta (A) and the pulmonary artery (PA).

With an Amplatz catheter, we came close to the ostium of this vessel. Upon injection, there appeared to be significant proximal stenosis or kinking of the vessel. However, it was challenging to visualize. In order to get better visualization of the anatomy of the right coronary artery, CT cardiac angiography was advised, which showed a large dominant right coronary artery arising from the left cusp just anterior to the left main coronary artery coursing between the aorta and pulmonary arterial trunk (Figure 2).

The initial few centimeters of the right coronary artery between the great vessels appeared to be significantly compromised at the very ostium and proximal portion, probably responsible for the small myocardial infarction. There was mild calcification in the mid-right coronary artery without significant obstruction. The patient was taken to the operating room on the weekend and performed an open-heart saphenous vein bypass involving the right coronary artery. Her internal mammary artery was not used for grafting because it was very small and could create a severe competitive flow issue. Her postoperative course was uneventful with the patient being discharged after three days.

## Discussion

In 1948, White and Edwards described the anomalous origin of the right coronary artery as a rare congenital anomaly [2]. Kaku et al., in their study conducted between 1968 and 1994 in Japanese centers, determined the prevalence of this anomaly as 0.25% by evaluating 17731 patients undergoing coronary angiography [5]. In another study conducted on the white population, the prevalence was much lower; 0.026% [6]. The aberrant right coronary artery can take a different course after its origin from the left coronary sinus; its course can be preaortic, retrocardiac, retroaortic, intra-septal, precardiac (prepulmonary), and most commonly inter-arterial between the aorta and pulmonary artery. The inter-arterial variant is the most clinically significant. It is subjective to external compression by the surrounding great vessels and can lead to potentially dangerous complications such as myocardial infarction, arrhythmias, and even sudden cardiac death [7].

Risk factors for sudden cardiac death are ≤40 years of age, male sex, and performing a sporting activity. Most patients are treated with surgical revascularization [8]. Syncope is the most common symptom of sudden cardiac death and death can occur in 50% of these patients [9]. In 1992, Taylor et al. examined the heart specimens of 242 autopsies that had documented isolated coronary artery anomalies, in which they found the prevalence of anomalous origin of the right coronary artery from the left coronary cusp to be 20%, among them 25% died suddenly [10]. Similarly, in another study at the University of Padua, 1200 anatomic specimens were collected and examined from 1968 to 1996 [11]. They found a sudden death rate of 43% among individuals who demonstrated an anomalous origin of the right coronary artery. It was an incidental finding, in which these patients died of non-cardiac causes. This study highlights the diverse presentation of this particular anomaly.

An individual can be asymptomatic; however, they will always be at risk of sudden cardiac death, especially during exercise when the coronary artery is present in the inter-arterial course because it is more prone to obstruction by the great vessels due to high systolic pressure [11]. Due to its potential for catastrophic complications, it should be managed on time. Choice of treatment is controversial, and it varies with the anatomical variant of the coronary arterial anomalies, patient comorbidities, and physician expertise. In Japan, Kaku et al. treated 56 patients (mean age 55.9 years) with an anomalous origin of a coronary artery from the left sinus with β-blockers, calcium channel blockers, and nitrates. They found no sudden deaths related to the congenital anomaly during the subsequent five years of follow-up [5]. However, different approaches have been used in surgical revascularization, such as detachment of the right coronary artery from the left sinus and reimplanting it into the right sinus, bypass grafting surgery (as was performed in our patient), unroofing technique, and direct translocation of the pulmonary artery [3]. In our case, the patient opted for surgical revascularization therapy to relieve myocardial ischemia in order to further reduce the risk of future catastrophic complications.

## Conclusions

In this study, we presented a rare symptomatic case of a congenital coronary artery anomaly. Anomalous origin of right coronary artery from left cusp and its aberrant course between the great vessels places the vessel at risk of compression that could lead to myocardial infarction, arrhythmias, or sudden death. Our patient with this anomaly had non-ST-elevation myocardial infarction (STEMI) and underwent coronary artery bypass surgery in a timely manner to relieve the ischemia, preventing the dire consequence of this coronary arterial anomaly.
